# Cortisol Regeneration in the Fetal Membranes, A Coincidental or Requisite Event in Human Parturition?

**DOI:** 10.3389/fphys.2020.00462

**Published:** 2020-05-25

**Authors:** Wang-Sheng Wang, Chun-Ming Guo, Kang Sun

**Affiliations:** ^1^Center for Reproductive Medicine, Ren Ji Hospital, School of Medicine, Shanghai Jiao Tong University, Shanghai, China; ^2^Shanghai Key Laboratory for Assisted Reproduction and Reproductive Genetics, Shanghai, China; ^3^School of Life Sciences, Yunnan University, Kunming, China

**Keywords:** 11β-HSD1, collagen, prostaglandins, glucocorticoids, fetal membranes

## Abstract

The fetal membranes are equipped with high capacity of cortisol regeneration through the reductase activity of 11β-hydroxysteroid dehydrogenase 1 (11β-HSD1). The expression of 11β-HSD1 in the fetal membranes is under the feedforward induction by cortisol, which is potentiated by proinflammatory cytokines. As a result, the abundance of 11β-HSD1 increases with gestational age and furthermore at parturition with an escalation of cortisol concentration in the fetal membranes. Accumulated cortisol takes parts in a number of crucial events pertinent to the onset of labor in the fetal membranes, including extracellular matrix (ECM) remodeling and stimulation of prostaglandin output. Cortisol remodels the ECM through multiple approaches including induction of collagen I, III, and IV degradation, as well as inhibition of their cross-linking. These effects of cortisol are executed through activation of the autophagy, proteasome, and matrix metalloprotease 7 pathways, as well as inhibition of the expression of cross-linking enzyme lysyl oxidase in mesenchymal cells of the membranes. With regard to prostaglandin output, cortisol not only increases prostaglandin E2 and F2α syntheses through induction of their synthesizing enzymes such as cytosolic phospholipase A2, cyclooxygenase 2, and carbonyl reductase 1 in the amnion, but also decreases their degradation through inhibition of their metabolizing enzyme 15-hydroxyprostaglandin dehydrogenase in the chorion. Taking all together, data accumulated so far denote that the feedforward cortisol regeneration by 11β-HSD1 in the fetal membranes is a requisite event in the onset of parturition, and the effects of cortisol on prostaglandin synthesis and ECM remodeling may be enhanced by proinflammatory cytokines in chorioamnionitis.

## Introduction

**FIGURE 1:**
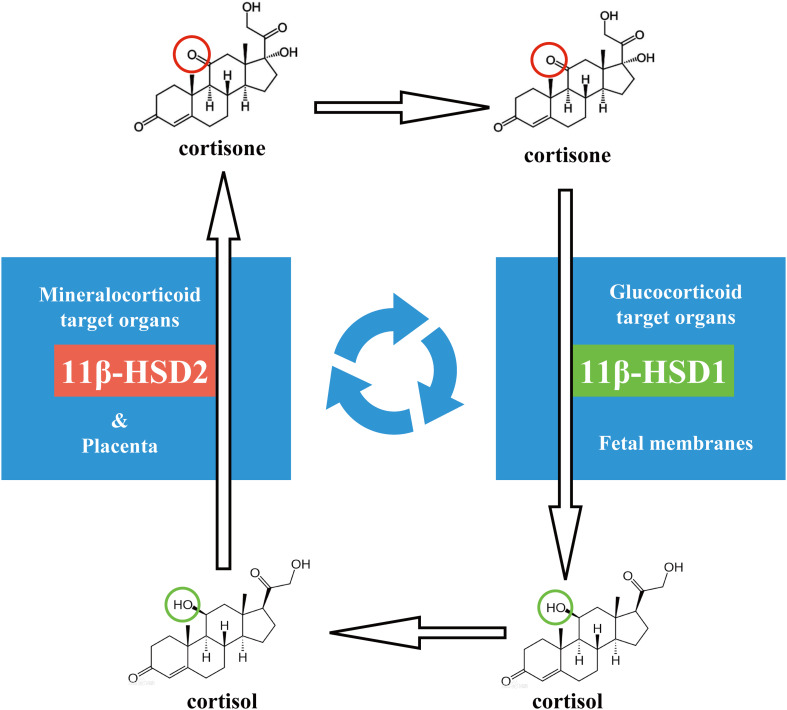
Recycle of cortisol and cortisone between placenta and fetal membranes in human pregnancy.

Glucocorticoids are essential for life, and it regulates a variety of important cardiovascular, metabolic, and immunologic functions in the maintenance of homeostasis (Schmid et al., 1995; Beato and Klug, 2000; Rhen and Cidlowski, 2005). Cortisol is the most important endogenous glucocorticoid in humans. The *de novo* synthesis of cortisol from cholesterol takes place primarily in the zona fasciculata of the adrenal cortex (Miller and Auchus, 2011). After secretion into the circulation, most of cortisol is bound by corticosteroid-binding protein (CBG) and to a lesser extent by albumin (Bae and Kratzsch, 2015; Meyer et al., 2016). There is approximately only 5 to 10% of cortisol that remains free in the circulation, which is important for the actions of cortisol as only the free fraction of cortisol is biologically active (Lewis et al., 2005). In compensation, glucocorticoid target organs develop a way to enhance cortisol concentrations within the cells through regeneration of cortisol by 11β-hydroxysteroid dehydrogenase 1 (11β-HSD1) (Chapman et al., 1997; Tomlinson et al., 2004; Chapman et al., 2013; Morgan et al., 2014). 11β-HSD1 is a microsomal reductase catalyzing the regeneration of cortisol from biologically inactive 17α-hydroxy-11-dehydrocorticosterone (cortisone), which derives mostly from the oxidase action of 11β-HSD2 in the mineralocorticoid target organs (Figure 1; Tannin et al., 1991; Albiston et al., 1994; Chapman et al., 2013). 11β-HSD2 is a counterpart enzyme of 11β-HSD1 and functions in an opposite way to 11β-HSD1 converting biologically active cortisol to inactive cortisone (Figure 1). Because 11β-HSD2 does not metabolize aldosterone, 11β-HSD2 is utilized by the mineralocorticoid target organs as a pre-receptor gate to ensure the indiscriminating mineralocorticoid receptor being occupied only by aldosterone but not by cortisol (White et al., 1997a,b,c). This differential expression pattern of 11β-HSD1 and 11β-HSD2 in glucocorticoid and mineralocorticoid target organs is developed perfectly to ensure the efficiency of cortisol’s actions and the specificity of aldosterone’s actions in their respective target organs.

In pregnancy, the placenta is responsible for nourishing and protecting the fetus as well as maintaining pregnancy by producing a plethora of hormones and immune factors. Attached to the edge of the discoid placenta is the atrophied chorionic villi, also known as the smooth chorion or chorion leave, which fuses with the amniotic membrane extended from the fetal surface of the placenta, and together they form the reflected fetal membranes (Leiser and Kaufmann, 1994; Ferner and Mess, 2011). The fetal membranes not only enclose the fetus bathed in the amniotic fluid but also become a source of initiating signals for parturition toward the end of gestation (Okazaki et al., 1981; Myatt and Sun, 2010; Menon, 2016; Wang et al., 2018; Menon and Moore, 2020). Like the specific distribution of 11β-HSD1 and 11β-HSD2 in glucocorticoid and mineralocorticoid target organs, the distribution of 11β-HSD1 and 11β-HSD2 in the placenta and fetal membranes also adopts a unique tissue-specific pattern (Sun et al., 1997; Yang et al., 2016). Although the placenta is not a typical mineralocorticoid target organ, it boasts abundant 11β-HSD2 but scarce 11β-HSD1 (Albiston et al., 1994; Sun et al., 1997; Yang et al., 2016). It is known that 11β-HSD2 in the placenta functions as a glucocorticoid barrier by inactivating maternal cortisol to cortisone so that the fetus can be protected from the growth-restricting effects of excessive maternal glucocorticoids (Osinski, 1960; Burton and Waddell, 1999; Drake et al., 2007). This function of 11β-HSD2 in the placenta is substantiated by its distinct distribution in the syncytiotrophoblast, the outmost layer of placental villi that immerse directly in the maternal blood (Krozowski et al., 1995; Ni et al., 2009; Li et al., 2011, 2013; Zhang et al., 2015; Zuo et al., 2017). In contrast to the placenta, the fetal membranes express abundant 11β-HSD1 with barely detectable 11β-HSD2 (Sun et al., 1997), which can utilize cortisone derived from both maternal mineralocorticoid organs and the placenta to regenerate cortisol (Figure 1; Murphy, 1977, 1979). The expression of 11β-HSD1 in the fetal membranes increases with gestational age and further increases in parturition with its abundance atop all fetal tissues by the end of gestation (Murphy, 1977, 1981; Alfaidy et al., 2003). This cortisol-regenerating capacity of the fetal membranes is even regarded as a supplemental extra-adrenal source of glucocorticoids in pregnancy (Murphy, 1977, 1981; Tanswell et al., 1977). Why should the fetal membranes be equipped with such a unique cortisol-regenerating capacity in pregnancy? Given that the smooth chorion is actually atrophied chorion villi, it is plausible to question whether this cortisol regeneration activity of the fetal membranes is merely an irrelevant by-product of pregnancy or a finely mapped-out event, which is absolutely required toward the end of gestation. In this review article, we will try to answer these questions by summarizing data from our laboratory as well as others.

## Feedforward Cortisol Regeneration in the Fetal Membranes

The fetal membrane components differ in different species (Mess et al., 2003). Human fetal membranes comprised the amnion and smooth chorion. The smooth chorion is the outer layer that connects the maternal decidua and can be subdivided into a trophoblast layer and a connective tissue layer adjacent to the amnion. The amnion is composed of a single layer of amnion epithelial cells sitting on a basement membrane and a tough compact layer that contains abundant interstitial fibers and fibroblasts (Bourne, 1960; Parry and Strauss, 3rd., 1998; Wang et al., 2018). Studies of 11β-HSD1 in the fetal membranes of other species are sparse. However, 11β-HSD1 has been localized to the placenta in a number of species including rat, sheep, and baboon (Burton and Waddell, 1994; Pepe et al., 1996; Yang et al., 1997). Immunohistochemical staining of human fetal membranes shows that 11β-HSD1 distributes in whole membrane layers (Sun et al., 1997; Wang et al., 2012; Liu et al., 2016a). In the amnion, 11β-HSD1 distributes in both epithelial and fibroblast cells, whereas in the smooth chorion, 11β-HSD1 is localized to trophoblasts, as well as fibroblasts (Sun et al., 1997; Wang et al., 2012; Liu et al., 2016a). Notably, in contrast to the expression of 11β-HSD2 in trophoblasts of human placenta chorionic villi, trophoblasts of the smooth chorion express mainly 11β-HSD1 rather than 11β-HSD2 (Sun et al., 1997). These discrepancies are suggestive of a unique role of 11β-HSD1 rather than a by-product of atrophied villi in the fetal membranes.

Intriguingly, cortisol, despite being a product of 11β-HSD1, induces rather than inhibits 11β-HSD1 expression in both smooth chorion trophoblasts and amnion fibroblasts (Sun et al., 2002; Sun and Myatt, 2003; Li et al., 2006; Yang et al., 2007), thus setting up a positive feedback loop between cortisol regeneration and 11β-HSD1 expression in the fetal membranes. This feedforward expression pattern of 11β-HSD1 in the fetal membranes may account for its increasing expression with gestational age (Alfaidy et al., 2003) and its further increase at parturition (Liu et al., 2016a).

The actions of glucocorticoids and proinflammatory cytokines usually oppose each other at sites of inflammation. However, in the fetal membranes, proinflammatory cytokines induce the expression of 11β-HSD1 not only on their own, but also in synergy with glucocorticoids (Sun and Myatt, 2003; Li et al., 2006; Lu et al., 2019c). Given that inflammation is a common cause of both term and preterm birth, and overproduction of proinflammatory cytokines is a common feature of inflammation (Bollapragada et al., 2009; Menon et al., 2010; Romero et al., 2014; Singh et al., 2019), the synergy between glucocorticoids and proinflammatory cytokines in the induction of 11β-HSD1 expression is particularly noteworthy because this synergy is very likely to generate even more cortisol under conditions of chorioamnionitis. These distinct features of 11β-HSD1 expression in the fetal membranes denote again that the expression of 11β-HSD1 in the fetal membranes may be a requisite event in the end of normal gestation and may even be more intriguing in the condition of chorioamnionitis. In non-gestational tissues, proinflammatory cytokines have also been shown to induce 11β-HSD1 expression either on its own (Tomlinson et al., 2001; Yong et al., 2002; Ignatova et al., 2009; Esteves et al., 2014) or in synergy with glucocorticoids (Rae et al., 2004; Kaur et al., 2010), which is regarded as a self-restraining mechanism to avoid over immune responses in inflammation. Is cortisol regeneration by 11β-HSD1 in the fetal membranes simply a self-restraining mechanism to contain inflammation or does it mean more than an anti-inflammatory role in pregnancy? Convincing evidence has accumulated that glucocorticoids derived from fetal adrenal glands can trigger parturition in a number of animal species (Anderson et al., 1975; Flint et al., 1978; First and Bosc, 1979; Morrison et al., 1983; Fowden et al., 2008). However, adrenal glands of human fetus produce mainly dehydroepiandrosterone sulfate (DHEAS) rather than glucocorticoids (Mesiano and Jaffe, 1997; Ishimoto and Jaffe, 2011). This feature of human fetal adrenal glands may also explain why systemic administration of synthetic glucocorticoids such as betamethasone apparently does not induce labor (Craft et al., 1976). This is probably due to the strong negative feedback of synthetic glucocorticoids on the fetal hypothalamic–pituitary–adrenal axis, which may result in diminished production of both DHEAS and cortisol when synthetic glucocorticoids pass through the placenta and enter the fetal circulation. Because DHEAS is a precursor for estrogen synthesis in the placenta, diminished DHEAS will lead to reduced estrogen production (Ogueh et al., 1999), which is essential for preparation of the myometrium for contraction (Mesiano, 2001). Therefore, it is very likely that the feedforward cortisol regeneration by 11β-HSD1 in the fetal membranes is a compensatory mechanism in parturition for the insufficient cortisol synthesis by fetal adrenal glands in humans. In other words, local regeneration of cortisol in the fetal membranes is probably more important than cortisol derived from fetal adrenal glands in human parturition. This notion is endorsed by increased abundance of glucocorticoid receptor (GR) in the fetal membranes at parturition (Sun et al., 1996), suggesting that cortisol regenerated by 11β-HSD1 in the fetal membranes has enhanced local actions pertinent to human parturition.

## Role of Cortisol Regeneration in the Rupture of Fetal Membranes

During the gestational period, a tough and tensile amniotic sac is required for holding and protecting the fetus bathed in the amniotic fluid. The tensile strength of the fetal membranes is believed to derive mainly from the rich content of collagenous fibers in the compact layer of the amnion (Bourne, 1962; Parry and Strauss, 3rd., 1998; Oyen et al., 2006). Needless to say, the fetal membranes should break for the delivery of fetus at parturition, and rupture of membranes can, in turn, promote labor, suggesting that the fetal membranes are a source of labor initiating signals. As a matter of fact, the fetal membranes are indeed among the gestational tissues that give rise to signals leading to parturition (Parry and Strauss, 3rd., 1998; Menon, 2016; Menon et al., 2016). These multiple source signals may need to work in a coordinating manner to start labor at term. However, intensified signals from the fetal membranes as elicited by membrane rupture or infection can sometimes even start labor alone no matter at term and preterm, which highlights the importance of membrane rupture in parturition. In line with the rupture of membranes, the fetal membranes become increasingly weak toward the end of gestation. Our serial studies indicate that local regeneration of cortisol is an important attributor to membrane weakening and rupture (Liu et al., 2016a; Wang et al., 2016, 2019a; Mi et al., 2017, 2018).

The initial inspiration that has encouraged us to investigate whether cortisol regeneration is involved in membrane rupture comes from the side effects of clinical usage of glucocorticoids. Based on their potent anti-inflammatory properties, synthetic glucocorticoids are widely prescribed to treat a variety of acute and chronic inflammatory conditions (Rhen and Cidlowski, 2005). Among the side effects of glucocorticoids, skin atrophy is frequently encountered (Sterry and Asadullah, 2002; Schoepe et al., 2006). Fibroblasts have been shown to be the target cells of glucocorticoids for this side effect (Nuutinen et al., 2001; Oishi et al., 2002; Schoepe et al., 2006). It has been demonstrated that glucocorticoids reduce tensile strength and elasticity of the skin by decreasing the synthesis of extracellular matrix (ECM) proteins and increasing their turnover in fibroblasts (Cutroneo et al., 1975, 1981; Shull and Cutroneo, 1986; Nuutinen et al., 2001; Oishi et al., 2002; Boudon et al., 2017). For the same reason, glucocorticoids are being used topically to prevent excessive scar formation (Berliner et al., 1967; Jalali and Bayat, 2007). These clinical observations have prompted us to envisage that the feedforward cortisol regeneration in the fetal membranes may be related to ECM remodeling for membrane rupture.

It is now known that the tensile strength of the amnion is largely attributed to collagen I and III contents in the compact layer (Bourne, 1960; Malak et al., 1993; Bryant-Greenwood, 1998). Abundance of collagen I and III in the amnion decreases after the middle trimester to a nadir at term (Skinner et al., 1981; Casey and MacDonald, 1996; Hampson et al., 1997). In preterm premature rupture of membranes, a common cause of preterm labor, collagen content is further decreased (Skinner et al., 1981; Hampson et al., 1997; Stuart et al., 2005). These findings suggest that decreased collagen abundance characterizes the process of membrane rupture. We investigated whether cortisol regenerated in the fetal membranes is involved in the reduction of collagens in cultured primary human amnion fibroblasts, which are the major source of ECM proteins in the amnion compact layer (Casey and MacDonald, 1996). We have demonstrated that cortisol decreases the abundance of collagen I and III protein in a concentration-dependent manner with no effects on their mRNA abundance in amnion fibroblasts. Further mechanistic studies have revealed that cortisol decreases collagen I and III protein abundance by activating the autophagic and proteasome pathways, respectively (Mi et al., 2017, 2018). In addition to collagen I and III, collagen IV is essential for the maintenance of epithelial basal membrane as well as for the ECM structural protein assembling in the amnion (Malak et al., 1993; Bryant-Greenwood, 1998). For this reason, collagen IV is known as another crucial determinant of membrane integrity. By using human amnion fibroblasts, we have found that cortisol also causes the breakdown of collagen IV in a concentration-dependent manner through drastic induction of the expression of matrix metalloprotease 7 (MMP-7), also known as matrilysin, *via* activation of the AP-1 transcription factor (Wang et al., 2019a). These inhibitory effects of cortisol on collagen I, III, and IV have shown to be GR-mediated effects and have been confirmed in amnion tissue explant experiments. These findings are also substantiated by concurrent increases in cortisol, 11β-HSD1, MMP-7, and markers for autophagy and decreases in collagen I, III, and IV in the amnion tissue collected from spontaneous labor with membrane rupture (Liu et al., 2016a; Mi et al., 2017, 2018; Wang et al., 2019a, b).

The tensile strength of the amnion is determined not only by collagen abundance, but also by the degree of their cross-linking. Cross-linked collagens become tough and resistant to the breakdown by MMPs (Vater et al., 1979). It is now known that the cross-linking of collagens is catalyzed by lysyl oxidase (LOX), a copper-dependent amine oxidase (Kagan and Trackman, 1991). It has been shown that LOX protein and enzyme activity decrease dramatically in the amnion with advancing gestational age and further decrease in spontaneous labor with membrane rupture (Casey and MacDonald, 1997; Liu et al., 2016a, b). We have demonstrated that both cortisol and cortisone inhibit LOX expression in human amnion fibroblasts, and the effect of cortisone is abolished with inhibition of 11β-HSD1 (Liu et al., 2016a). Again, these effects have proved to be mediated by GR and have been replicated in amnion tissue explant experiments.

**FIGURE 2:**
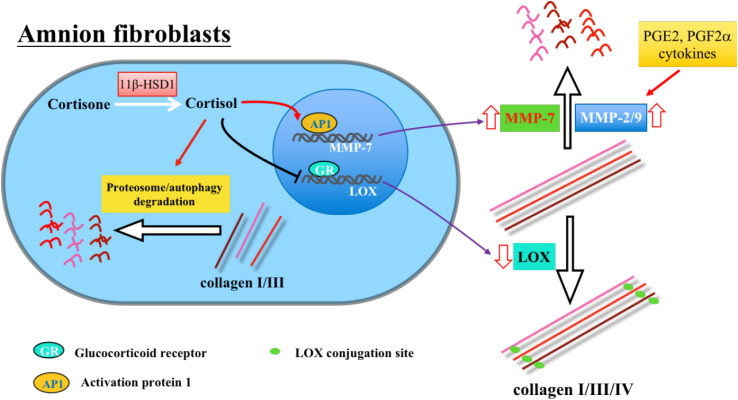
Extracellular matrix (ECM) remodeling effects of cortisol regenerated by 11β-HSD1 in the fetal membranes.

In addition to the effects mediated directly by GR, an alternative mechanism is also present in ECM remodeling effects of cortisol. It has been shown that serum amyloid A1 (SAA1), an acute phase protein produced primarily by the liver, could be produced locally in the fetal membranes (Li et al., 2017), where SAA1 exerts extensive ECM remodeling effects including induction of MMP-1, MMP-2, MMP-8, MMP-9, and MMP-13; inhibition of LOX-like 1; and evoking collagen degradation (Wang et al., 2019b, c). Moreover, SAA1 can induce the expression of 11β-HSD1, and cortisol can in turn stimulate the expression of SAA1 (Lu et al., 2019b, c), thus formulating a mutual reinforcing mechanism in the production of SAA1 and cortisol in the fetal membranes. These findings are suggestive of existence of an alternative mechanism through induction of SAA1 to remodel the ECM structure by cortisol in the fetal membranes. Taken together, all these findings of cortisol’s actions on collagens, LOX, and MMP-7 in human amnion fibroblasts are supportive of the view that cortisol regenerated by 11β-HSD1 plays an important role in the ECM remodeling for membrane rupture at parturition (Figure 2).

## Role of Cortisol Regeneration in the Stimulation of Prostaglandin E2 and F2α Output in the Fetal Membranes

Prostaglandins, particularly prostaglandin E2 (PGE2) and prostaglandin F2α (PGF2α), play crucial roles in human parturition (Challis et al., 1997; Romero et al., 2014). They increase myometrium contractility, ripen the cervix, and promote fetal membrane rupture (Challis et al., 1997; Romero et al., 2014). Although all gestational tissues including fetal membranes and maternal decidua/myometrium are virtually capable of PGE2 and PGF2α synthesis, the fetal amnion and maternal decidua/myometrium are recognized to synthesize the most PGE2 and PGF2α, respectively (Duchesne et al., 1978; Mitchell et al., 1978; Okazaki et al., 1981; Rehnstrom et al., 1983). In the amnion, the capacity of PGE2 synthesis in interstitial fibroblasts is approximately 5 times more than the capacity in epithelial cells (Sun et al., 2003). In addition, amnion fibroblasts are also capable of synthesizing PGF2α, although not as much as PGE2 (Guo et al., 2014). As narrated above, glucocorticoids are the most widely used class of anti-inflammatory drugs with prominent inhibitory effects on the synthesis of proinflammatory cytokines, as well as prostaglandins (Rhen and Cidlowski, 2005). These inhibitory effects of glucocorticoids on prostaglandin synthesis are known to be executed mostly through inhibition of cyclooxygenase 2 (COX-2) expression, the rate-limiting enzyme in prostaglandin synthesis (Goppelt-Struebe et al., 1989; Lasa et al., 2001; Lim et al., 2014). However, it has been noted for a long time that there are parallel increases in cortisol and prostaglandin levels in the maternal circulation toward the end of human gestation, and this apparent contradiction is described as a gestational paradox (Casey et al., 1985). Several groups have confirmed that this paradoxical phenomenon holds true in human amnion fibroblasts (Potestio et al., 1988; Zakar et al., 1995; Blumenstein et al., 2000; Sun et al., 2003, 2006). It has been shown that both cortisol and synthetic glucocorticoids stimulate rather than inhibit the expression of COX-2 in human amnion fibroblasts. Moreover, glucocorticoids also induce the expression of cytosolic phospholipase A2 (cPLA2) in human amnion fibroblasts (Sun et al., 2003; Guo et al., 2008, 2010). Cytosolic phospholipase A2 catalyzes the formation of arachidonic acid, a rate-limiting substrate in prostaglandin synthesis, from membrane phospholipids. The mechanism underlying the paradoxical induction of cPLA2 and COX-2 by glucocorticoids is fascinating because glucocorticoids inhibit the expression of proinflammatory cytokines in amnion fibroblasts at the same time. We set out to delineate this paradoxical mechanism and it turned out to be a very complicated mechanism. It is revealed that glucocorticoids induce cPLA2 and COX-2 expression through stimulation of the cAMP/PKA pathway with subsequent activation of multiple transcription factors including CREB and STAT3, and so on (Zhu et al., 2009; Guo et al., 2010; Wang et al., 2015; Lu et al., 2017, 2019a). Interestingly, the classical inflammatory transcription factor, nuclear factor κB, is nevertheless inhibited by glucocorticoids (Guo, 2010), which is responsible for the inhibition of proinflammatory cytokine expression in human amnion fibroblasts, a situation resembling most of non-gestational tissues.

As depicted above, the amnion is also capable of synthesizing PGF2α, although not as much as PGE2 (Guo et al., 2014). There are multiple pathways for PGF2α synthesis in amnion fibroblast. In addition to PGF synthase (PGFS)–catalyzed formation of PGF2α from PGH2, PGF2α can also be converted from PGE2 by the enzyme carbonyl reductase 1 (CBR1) (Ziboh et al., 1977). We have found that cortisol significantly induces the synthesis of PGF2α through induction of CBR1 but not PGFS in amnion fibroblasts (Sun et al., 2003; Guo et al., 2014). This induction of CBR1 by cortisol was revealed to be GR-mediated enhancement of CBR1 transcription (Guo et al., 2014).

**FIGURE 3:**
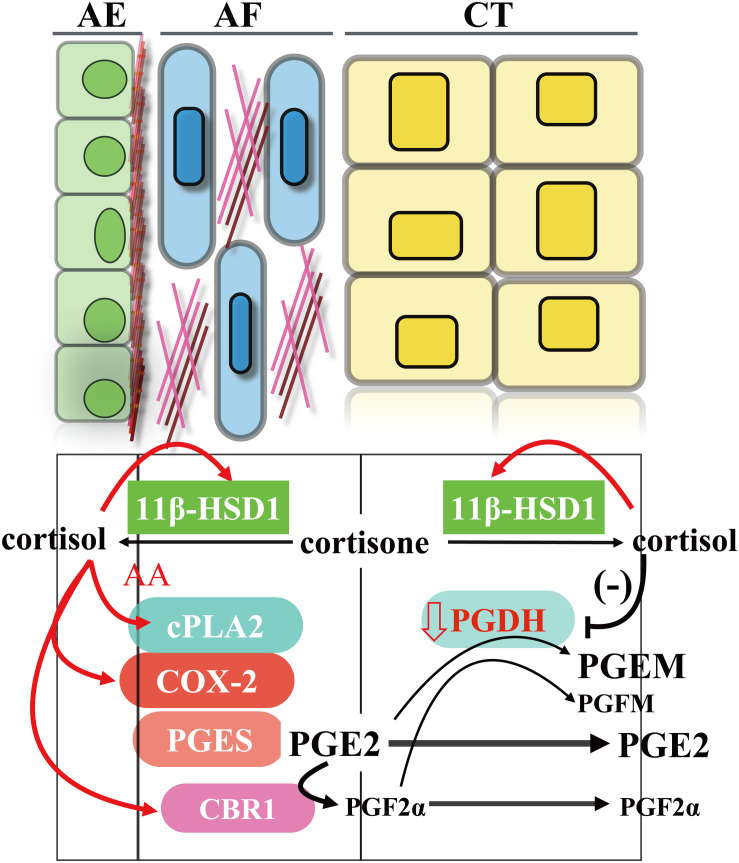
Induction of PGE2 and PGF2α output by cortisol regenerated through 11β-HSD1 in human fetal membranes. AE, amnion epithelial cells; AF, amnion fibroblasts; CT, smooth chorion trophoblasts; AA, arachidonic acid.

During the gestational period, there is abundant expression of prostaglandin degrading enzyme 15-hydroxyprostaglandin dehydrogenase (PGDH) in trophoblasts of the smooth chorion, which is known as a prostaglandin barrier (Cheung et al., 1992; Johnson et al., 2004). PGDH catalyzes NAD^+^-linked oxidation of 15 (S)-hydroxyl group of prostaglandins resulting in inactivation of their biological activities (Tai et al., 2002). Therefore, PGE2 and PGF2α synthesized in the amnion are mostly blocked from reaching the uterus by this barrier. Studies have shown that the abundant expression of PGDH in chorion trophoblasts is maintained mostly by progesterone (Challis et al., 1999; Patel and Challis, 2002). However, this maintenance is eventually undermined by increasing concentrations of cortisol derived from the feedforward regeneration through 11β-HSD1 (Patel et al., 1999a, b; Patel and Challis, 2002). The involvement of 11β-HSD1 is supported by findings that inhibition of PGDH by cortisone is reversed by 11β-HSD1 inhibitor (Patel et al., 1999b). It has been suggested that accumulating cortisol may compete with progesterone for progesterone receptor, thereby attenuating the maintaining effect of progesterone on PGDH expression and leading to progressively undermined prostaglandin barrier in the smooth chorion at term (Patel et al., 2003). Like the situation of cPLA2 and COX-2 induction by glucocorticoids, the inhibition of PGDH by glucocorticoids in the smooth chorion is also a kind of paradox, which is in marked contrast to the induction of PGDH expression by glucocorticoids in most of non-gestational tissues (Xun et al., 1991; Tong and Tai, 2005). All these findings of glucocorticoids on the induction of cPLA2/COX-2/CBR1 in amnion fibroblasts and inhibition of PGDH in chorion trophoblasts are supportive of a role of cortisol regeneration in the stimulation of PGE2 and PGF2α output either by induction of their synthesis or by inhibition of their degradation in the fetal membranes (Figure 3).

## Summary and Perspectives

In pregnancy, there is increasing cortisol regeneration by 11β-HSD1 toward the end of gestation. 11β-HSD1 is expressed in virtually all cell types in the fetal membranes. Although the smooth chorion of the fetal membranes is considered as atrophied chorionic villi, the fetal membranes including the smooth chorion are endocrinologically active in pregnancy. Cortisol regeneration by 11β-HSD1 is one of such endocrine activities. Cortisol regenerated by 11β-HSD1 is involved not only in ECM remodeling for membrane rupture but also in the upregulation of PGE2 and PGF2α outputs. Both are requisite events for the onset of parturition. In addition to the actions described in this review, it remains to be uncovered whether cortisol regenerated by 11β-HSD1 in the fetal membranes possesses other unknown actions pertinent to the onset of labor. Another interesting issue remains to be clarified is the apparent contradictions between the concurrent induction of prostaglandins and inhibition of proinflammatory cytokines by glucocorticoids in the fetal membranes. Because both prostaglandins and proinflammatory cytokines are prolabor factors involved in ECM remodeling and uterine contractile activities (Keelan et al., 2003), it would be interesting to understand how these actions of cortisol on prostaglandins and proinflammatory cytokines are balanced in the fetal membrane at parturition, particularly in the situation of chorioamnionitis. Nevertheless, these effects of cortisol on ECM remodeling and prostaglandin output might be even enhanced in chorioamnionitis, given the potentiation of cortisol regeneration by proinflammatory cytokines. These apparent contradictions may represent a unique feature how glucocorticoids work locally in the fetal membranes in the promotion of labor. Simultaneous inhibition of proinflammatory cytokines and stimulation of prostaglandins by glucocorticoids may avoid deleterious effects of proinflammatory cytokines on the fetus on the one hand but save the labor promoting effects of prostaglandins on the other hand. Finally, it would be helpful to find a suitable animal model to replicate those findings in humans, such as the expression pattern of 11β-HSD1 across gestational age and those actions of glucocorticoids on ECM remodeling, prostaglandin synthesis, and degradation in the fetal membranes. What is more important is to test whether local artificial manipulation of 11β-HSD1 and GR expression in the fetal membranes can indeed change the course of gestation in the right animal model. Taking all together, we can conclude that cortisol regeneration in the fetal membranes is not a coincidental but a requisite event in parturition.

## Author Contributions

KS conceived the idea. KS, W-SW, and C-MG contributed to manuscript writing and figure preparation.

## Conflict of Interest

The authors declare that the research was conducted in the absence of any commercial or financial relationships that could be construed as a potential conflict of interest.
